# A Scoping Review of Drug Epidemic Models

**DOI:** 10.3390/ijerph19042017

**Published:** 2022-02-11

**Authors:** Wei Wang, Sifen Lu, Haoxiang Tang, Biao Wang, Caiping Sun, Pai Zheng, Yi Bai, Zuhong Lu, Yulin Kang

**Affiliations:** 1 Institute of Environmental Information, Chinese Research Academy of Environmental Sciences, Beijing 100012, China; weiwang@craes.org.cn (W.W.); wang.biao@craes.org.cn (B.W.); suncp@craes.org.cn (C.S.); 2 Precision Medicine Key Laboratory of Sichuan Province and Precision Medicine Center, West China Hospital, Sichuan University, Chengdu 610041, China; sifenlu2017@163.com; 3 Department of Biomedical Engineering, College of Engineering, Peking University, Beijing 100871, China; thx1993@pku.edu.cn; 4 Department of Occupational and Environmental Health Science, School of Public Health, Peking University, Beijing 100871, China; zhengpai.mail@gmail.com; 5 Department of Epidemiology and Biostatistics, School of Public Health, Peking University, Beijing 100871, China; 15652249516@163.com; 6 State Key Lab of Bioelectronics, National Demonstration Center for Experimental Biomedical Engineering Education, School of Biological Science and Medical Engineering, Southeast University, Nanjing 210096, China; zhlu@seu.edu.cn

**Keywords:** drug epidemic model, mathematical epidemiology, nonlinear dynamic systems

## Abstract

The phenomenon of drug epidemics has been a global issue in the past decades, causing enormous damages to the physical and mental health of drug users and social well-being. Despite great efforts to curb drug epidemics at the governmental or social level, the total number of drug users has still been on the rise in recent years, along with illicit production and trafficking around the world. Inspired by dynamical epidemic models of infectious disease, a flourishment of promising results has been observed in the exploration of drug epidemic models. In this review, we aim to provide a scoping review of all existing drug epidemic modeling studies, and it has been shown that most studies focused on analyses of theoretical behaviors of the model systems, lacking emphasis on practical applications in real settings. We found that the drug epidemic models were characterized by a longer time scale, no incubation period, no significant prevention vaccines interfered, and population specificity. This review could assist policymakers and public health workers in gaining deeper insights into modeling tools, and help modelers improve their works, thus narrowing gaps between mathematical epidemiology and public health studies.

## 1. Introduction

The phenomenon of drug abuse, which involves the consumption of illicit drugs or narcotics and nonmedical use of prescription drugs, has become one of the major issues threatening the safety and sustainability of human society in the 21st century. According to the 2019 World Drug Report released by United Nations Office on Drugs and Crime (UNODC), approximately 271 million people, which constituted 5.5% of the global working-age population (15–64 years), had used drugs in 2016 [1]. From a historical perspective, the current figure is 30% higher than it was in 2009 [1,2]. In terms of drug type, opioids remained the most lethal group, which resulted in around 66% of overdose-related deaths worldwide in 2017 [1]. Besides, the level of manufacture and trafficking of conventional drugs such as cocaine and cannabis remained high, and that of synthetic drugs such as methamphetamine and MDMA soared in recent years. Some 35 million people suffered from drug use disorders and required treatment around the world, and the death toll attributed to drug use totaled 585,000 in 2017 [1]. All the evidence above aroused the need for global attention to the problem of drug abuse, as well as targeted intervention to address this issue.

A mathematical model has long been an effective tool in the area of public health, and extensive research has been conducted in terms of drug users and drug-using behaviors. In the 1970s, the analogy between heroin use and communicable disease was proposed, which verified the validity and utility of the epidemiologic approach to studying heroin use [3,4,5]. This paved the way for the utilization of compartmental dynamic models, which turned out to be a powerful tool in the field of infectious disease, in the research of drug users [6,7]. Several early research projects have emerged in the past decades which adopted the framework of compartmental dynamic models until in 2006, a classic three-compartment dynamical model was proposed by White and Comiskey [8]. The authors utilized ordinary differential equations (ODE) systems and made calculations on several key aspects of the model, including basic reproduction number (R_0_), drug-free equilibrium and endemic equilibrium. Later on, with the aid of development in nonlinear dynamic systems and computer-assisted simulation tools, plenty of research has sprung up worldwide, adding to this field of drug epidemic model. Through appropriate modeling and simulation, drug epidemic models shall be able to predict the scale and trend of drug abuse, and aid policymakers in evaluating the effect of intervention strategies by means of sensitivity analysis [9].

Given the vital importance and potential for wide applications of drug epidemic models outlined above, it is surprising to find that systematic reviews on this topic can barely be found [10]. Hence it is our goal to identify all existing research in the field of drug epidemic models, including pioneering studies based on compartmental dynamic models. In detail, we mainly focused on the evolution of basic assumptions and group definitions of the modeling studies, which might be in the interest of epidemiologists and policymakers. Besides, through exhaustive characterization of the modeling techniques and in-depth discussions of potential defects, this review also aims to help modelers improve their work and narrow the existing gaps between mathematicians and public health workers in the field of mathematical epidemiology.

## 2. Methods

We conducted a scoping review of all published drug epidemic modeling studies based on compartmental dynamic models. Relevant studies which evaluated the evolution of the number or proportion of drug-related subpopulations utilizing drug epidemic models were all included for analysis. The adoption of the methodology of scoping review, which is a powerful tool aimed at rapidly mapping key concepts underpinning a research area and the main sources of evidence available, was mainly based on concerns about the limited number of research in this field as well as diversified modeling techniques under investigation [11]. Therefore, a systematic review following highly focused research questions seemed unrealistic and hence inappropriate for this objective.

### 2.1. Search Strategy

We searched the PUBMED/Medline and SCOPUS databases from inception to 1 April 2020 using the following search terms:

- (“Heroin” OR “Synthetic drug” OR “Methamphetamine”) AND
- (“Epidemic model” OR “Transmission model”)

### 2.2. Article Selection

Articles which met the following criteria were eligible for inclusion:

- Drug epidemic modelling studies based on compartmental dynamic models;
- Models based on mathematical tools including ordinary differential equations (ODE), partial differential equations (PDE), delayed differential equations (DDE), stochastic differential equations (SDE) and difference equations (DE) were all considered eligible;
- Models simply investigating HIV epidemic among populations including drug users, without any focus on drug epidemic, were excluded;
- Systematic reviews and articles not in English were excluded.

We used EndNoteX8 (Clarivate Analytics, New York, NY, USA) software to manage exported search results. After removing duplicates, we initially screened the titles and abstracts to identify all potentially eligible results. Subsequently, the full texts and supplementary information if necessary, of all remaining articles were read, further excluding articles that did not meet inclusion criteria. Finally, articles identified via other sources, including reference lists of included articles, which also met the inclusion criteria, were also considered for this review.

**Figure 1:**
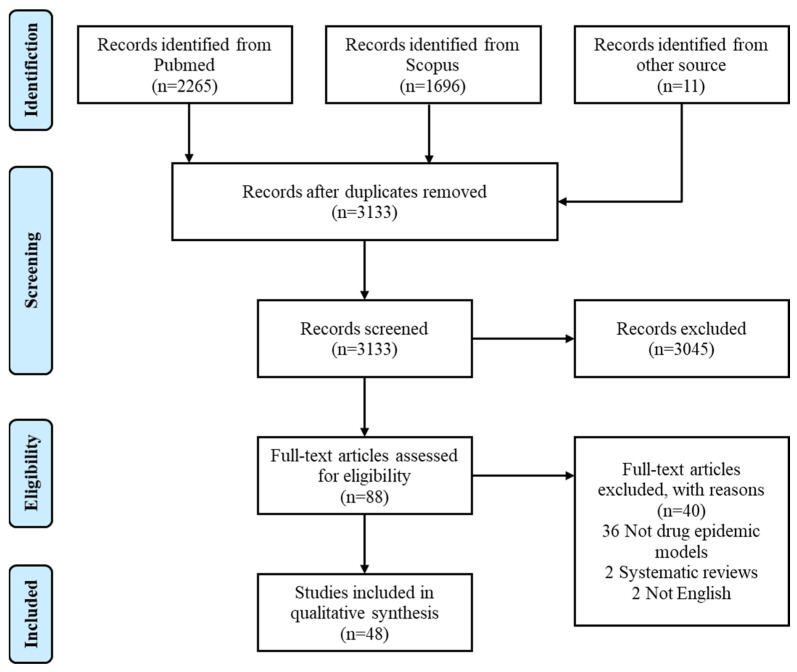
PRISMA diagram of study selection processes.

Our review was conducted in accordance with the Preferred Reporting Items for Systematic reviews and Meta-Analyses (PRISMA) guidelines, and the flow diagram of the inclusion and exclusion process was shown in Figure 1 [12].

### 2.3. Data Extraction

We extracted and tabulated the general information and model characteristics of all eligible articles, including year of publication, equation type, number of compartments, drug type, type of incidence function, intervention and scenario (if applicable).

## 3. Results

### 3.1. Characteristics of Studies

Our search criteria returned 3972 references, of which 3133 were unique records (Figure 1). Among 88 studies with full text assessed for eligibility, 48 articles met the selection criteria and were hence included for analysis. We tabulated the model characteristics and settings in Table 1. 23 of the 48 studies included were based on ODE modeling, 10 adopted PDE models (excluding delayed or stochastic PDE), eight used DDE models, five were SDE models and the remaining two studies took the form of DE models. 13 studies applied their models to real settings or scenarios, either choosing parameters pertaining to local conditions or fitting models to historical data. Among these studies, seven were set in Africa, four in Oceania or North America, one in Europe and one in Asia. In terms of drug type, there were 28 heroin epidemic models, 6 methamphetamine models, four synthetic drug models, two co-infection models and eight unspecified drug epidemic models.

**Figure 2:**
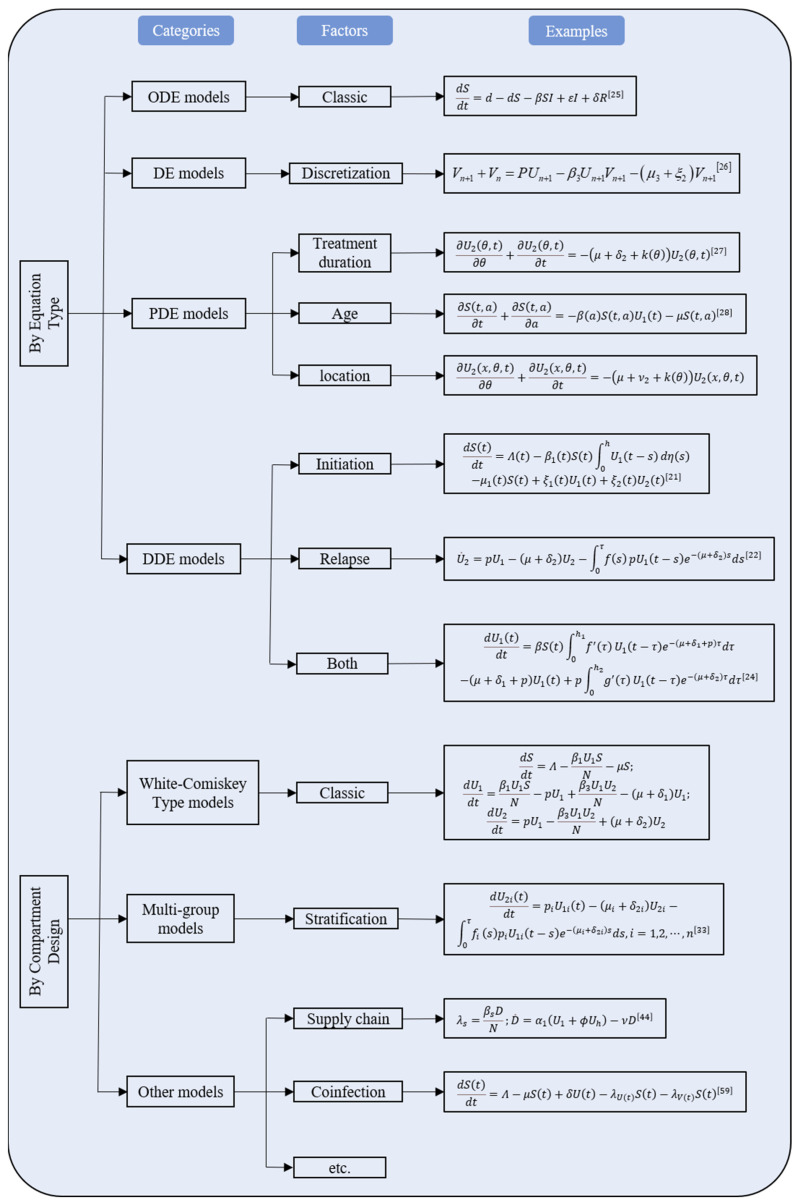
Mathematical expressions of all the drug epidemic models.

Other factors considered for classification included type of incidence function and intervention. An incidence function taking the formulation of $\frac{\beta SU}{N}$ is called standard incidence function, where β stands for effective contact ratio, S, U and N refer to the number of susceptible, drug user and total population [9]. Likewise, incidence functions with the formulation of βSU or βS are called bilinear incidence function and linear incidence function, respectively. All other complicated forms were referred to as complex incidence functions. 7 of the 48 included studies adopted standard incidence function, 20 adopted bilinear incidence function, 4 used linear incidence function and 17 adopted complex incidence function. Interventions could be in the form of compartment or parameter or both, where compartmental interventions denoted that the model included compartments of the drug user or patients under treatment, while parametric interventions implied that the study conducted sensitivity analysis in order to identify the most effective parameters to put the drug epidemic under control. The mathematical expressions of the drug epidemic models were summarized. (Figure 2).

Subsequently, we categorize the included articles into 4 groups according to their modeling frameworks and methodologies adopted and summarize the studies in chronological order.

### 3.2. Early Attempts of Drug Epidemic Model

In 1981, Hoppensteadt et al. proposed a combined model of drug interaction at the individual level and drug epidemic at the macro level [13]. They first modeled an individual’s response to a drug based on interactions between applied dosage and receptor sites in the body and used it subsequently as infectiousness in the drug epidemic model. The model, which divided the population into susceptibles and infectives, incorporated age dependency in infectiousness and cure rate. The authors also defined and analyzed a threshold value to determine the existence of the drug epidemic and identified certain key parameters for controlling it.

In 1997, Knolle combined compartmental models with a probabilistic framework while estimating incidence and prevalence of illegal drug use in Switzerland [14]. The study classified all drug users into two groups according to whether a user had been charged before and formulated two indicators as reflections of incidence. However, the assumption of exponential growth seemed unrealistic, and the prevalence estimates were conducted mainly by fitting probability distribution functions to historical data.

Almeder et al. proposed an age-structured drug initiation model in 2004 [15]. Under the assumption of a constant total population, the 2-group model was reduced to a single-state PDE system, which incorporated age-specificity through a complex initiation rate formulation. The initiation rate was assumed to be the product of three different factors: a basic age-specific rate, the influence of drug reputation and a prevention factor mirroring the effect of prevention programs. Numerical simulation results showed a pattern of oscillation in the unperturbed system, and the existence of such a “drug cycle” phenomenon depended mainly on some key parameters and boundary values. The study also evaluated the effect of prevention strategies via control models, providing deeper insights into the designing of age-structured prevention programs and optimal allocation of prevention expenditures.

A five-state model was presented by Caulkins et al. in 2007 to reproduce historical trends and make predictions of Australia’s drug epidemic [16]. The model was based on five linear difference equations, and divided the population according to drugs consumed, frequency of injection and possibility of escalation. The system displayed significant delay and inertia due to the nature of difference equations, however, the evaluation of three stylized interventions (primary prevention, supply control and harm reduction) still generated policy insights for reducing drug-related social costs.

Two similar studies were conducted by Caulkins et al. during 2009–2010 based on a two-state drug epidemic model [17,18]. Taking the percentage reduction in harmfulness of drug use as the control variable, the model aimed at simulating the effect of harm reduction policy adopted in different countries, and during various stages of the epidemic. The 2010 article modified the force of infection to allow for general formulations concerning virulence and initiation rate, as well as two coefficients of innovation and imitation, respectively. The studies proposed a primitive-type tipping point, which divided two equilibria under certain circumstances. Numerical simulations parameterized for Australian IDU and US cocaine showed that implementing harm reduction might dramatically increase both drug use and total harm, depending on the initial value of drug prevalence. These studies provided inspirations for solving the debate between use reduction and harm reduction, in that proponents of both sides shall explain the applicability of their policies in certain scenarios, instead of trying to find a universal solution to all relevant issues.

### 3.3. The White–Comiskey Model

The above-mentioned articles were mostly early attempts and their follow-up research in the field of drug epidemic models, which differed significantly from the rest of the articles reviewed. The watershed event between them was the publication of the classic White–Comiskey model in 2007, which acted as a landmark for a great deal of drug epidemic modeling studies to follow [8].

Under the background of heroin prevalence in Ireland, the authors aimed at introducing a universal heroin epidemic model which incorporated the treatment-relapse cycle in the drug-using career. The population of interest were all individuals aged 15–64, and were divided into three compartments: susceptible individuals, drug users not in treatment and drug users in treatment. The model assumed constant total population and adopted standard incidence function in new initiates and relapses.

Some key concepts were borrowed from compartmental dynamic models of infectious diseases, including basic reproduction number ($R_{0}$), drug-free equilibrium (DFE), endemic equilibrium (EE) and backward bifurcation. Basic reproduction number $R_{0}$, which was defined as the number of secondary cases resulting from an infected individual (or drug user not in treatment in this case) introduced into a susceptible population, played as the threshold value for the drug epidemic. The condition of $R_{0}<1$ implied that a drug epidemic will not happen and was used for the proof of local stability of the DFE. The opposite case of $R_{0}>1$ implied the persistence of the drug epidemic and was involved in the proof of existence of EE. The special case of $R_{0}=1$ needed further investigation, where a backward bifurcation might occur, which means the existence of multiple EEs, and that $R_{0}$ needed to be lowered to a level far below one to eradicate the drug epidemic.

The study also conducted sensitivity analyses of $R_{0}$ to identify the most effective parameters to curb the drug epidemic. After ruling out the seemingly invariant demographic parameters, the study identified $\beta_{1}$, the probability of an individual becoming a drug user, as the most effective parameter. A simple deduction would be that prevention was indeed better than cure, and the significance of parameterization was also raised.

The White–Comiskey model has definitely paved the way for subsequent models of the drug epidemic, and its influence has gone far beyond the original Irish setting. A secondary analysis of this model was conducted by Mulone et al. in 2009 [19]. The authors preserved the assumption of constant total population but removed the restraint of constant inflow rate. The steady states of the model were extensively discussed in this article, and the unique EE was proved to be locally and globally asymptotically stable. In a subsequent study based on the Mulone model in 2011, the authors relaxed the assumption of constant total population, and replaced the standard incidence function with bilinear incidence function [20]. The existence and stability of the system equilibria were discussed, and numerical simulations were used to illustrate the dynamic behaviors of the system. Besides, simulation results of examples where impulsive input or stochastic perturbations were introduced to the original system were also displayed.

### 3.4. Modifications of the White–Comiskey Model

Plenty of studies have been motivated by the White–Comiskey model in the past decade, and various types of modifications were applied to the classic model. Some of the representative ones are summarized in the following sections.

#### 3.4.1. Models Based on DDE

In 2011, Samanta modified the classic White–Comiskey model into a nonautonomous heroin epidemic model with distributed delay [21]. Two threshold values were introduced for the permanence and extinction of the heroin epidemic, respectively. Given the nature of nonautonomous systems, the author defined the global asymptotic stability of the system instead of an equilibrium and derived sufficient conditions via the method of Lyapunov functional. Another heroin epidemic model with distributed time delay based on the White-Comiskey model was introduced by Liu et al. in the same year [22]. The delay, however, occurred during the relapse process to untreated drug use, which differed from the former study. Still, the stability of equilibria and permanence of the system were discussed, and results of the sensitivity analysis also showed that prevention efforts were most effective in controlling the spread of habitual drug use. This model was revisited by Huang et al. in 2013, and the global asymptotic stability of the EE was investigated using the direct Lyapunov method [23].

In addition, a heroin model with two distributed delays was put forward by Fang et al. in 2014 [24]. The model incorporated a progression-to-use delay as well as a relapse delay, and established conditions for the local and global asymptotic stability of the DFE and a unique EE. Moreover, sensitivity analysis also identified the transmission coefficient as the most effective parameter to intervene in the heroin epidemic, which shared similarities with the conclusions of the aforementioned studies.

#### 3.4.2. Models Based on ODE or DE

In 2014, Muroya et al. treated the behavior of “light drug” use as a sort of infectious disease and turned the White-–Comiskey model into a SERS epidemic model with graded cure and incomplete recovery rates [25]. The model assumed no drug-related deaths and allowed for flows of recovery from light drug users not in treatment and undergoing treatment back to the susceptible class. The authors discussed the eventual boundedness and permanence of the system and established the global stability of the equilibria through two types of Lyapunov functional techniques. Another study conducted by Abdurahman et al. in the same year modified the Samanta model into a discretized heroin epidemic model with delay [26]. By applying a nonstandard finite difference scheme, the model became a set of nonautonomous difference equations, and sufficient conditions of the global dynamics of the system were obtained for a special case of constant infectivity.

#### 3.4.3. Models Based on PDE

Following the early studies based on ODE systems, several following studies utilizing PDE systems have emerged, which rendered drug epidemic models higher complexity and applicability. In 2015, Fang et al. modified the classic White–Comiskey model into a PDE model with treat-age [27]. By accounting for the effect of treatment duration on relapse rate, the authors incorporated dual-dependence of time and treat-age into the model. The global dynamics of the system were acquired, though mathematical tools adopted differed significantly from those used for ODE models. The same research group proposed another PDE model in the same year, which involved the age-dependent susceptibility of the susceptible individuals [28]. Global dynamics of the model were also investigated, and the stability of the equilibria was proved to be totally determined by the basic reproduction number.

The complex incidence rate was integrated into a heroin epidemic model with age structure by Yang et al. in 2016 [29]. The model adopted a universal incidence function for initiation, and a duration-dependent relapse rate as well. Upon establishing sufficient conditions for the global dynamics of the model, the authors chose a piecewise relapse function and a saturation incidence function for illustration. Similar assumptions were adopted by another research by Djilali et al. in 2017 [30]. The model differed from the previous one in that a nonlinear incidence function in its very general form was considered, which was compatible for various function types, including a bilinear function, a Beddington–DeAngelis function or a separable product of two nonlinearities.

In 2019, Liu et al. considered an age-structured heroin epidemic model, where age dependency was incorporated into susceptibility, relapse rate and drug-related death or removal rate [31]. The global behaviors of the system were proved to be totally determined by the basic reproduction number, and optimal control strategies, as well as some special cases of the model formulation were discussed. In the following year, Duan et al. formulated a diffusive age-structured heroin epidemic model [32]. The model accounted for the influence of location on treatment rate, and the effect of treatment duration on relapse rate as well. Besides, spatial restrictions were applied, in that heroin users undergoing treatment had a fixed location. The authors established the threshold dynamics of the system and presented simulation results for verification.

#### 3.4.4. Multi–Group Models

Multi–group epidemic models divide the host population into several parallel groups according to their inherent qualities (e.g., gender, age, ethnicity, location, etc.), and have been extensively used to incorporate population heterogeneity. One of the first applications of this method in drug epidemic modeling studies turned up in 2016 when Liu et al. established a delayed multi-group heroin epidemic model based on Liu et al.’s DDE model [33]. The model divided the total population into several parallel groups and allowed for interactions between groups when formulating initiates and relapse rates. The authors adopted a nonlinear incidence function and completed the analysis of a sharp threshold property. In the same year, Yang et al. constructed a heroin epidemic model based on scale-free complex networks [34]. In addition to obtaining threshold dynamics of the system, the authors have shown the persistence of the heroin epidemic for sufficiently large networks. Another delayed multi-group heroin epidemic model in the heterogeneous population was considered by Liu et al. in 2016, where distributed delay was adopted for both initiates and relapse rates [35]. The authors finished proofs of the threshold dynamics through the graph-theoretic approach and Krichhoff’s matrix tree theorem and performed simulation studies for the case of two groups. In 2019, Wang et al. developed an age-structured multi-group heroin epidemic model based on Fang et al.’s model, which accounted for different levels of susceptibility and relapse rate in each group [36]. After rigorous investigation of the dynamical behaviors of the system, the authors also provided detailed illustrations for a special case of two groups, where gender was chosen as the factor for stratification.

#### 3.4.5. Models Based on SDE

The drug epidemic, as with the spread of infectious diseases, is inevitably influenced by unexpected events or social fluctuations, which correspond to stochastic perturbations in drug epidemic models. The stochastic epidemic model is a powerful tool to account for the effect of random disturbance on the spread of habitual drug use, and drug epidemic models based on SDE started to emerge in 2018, when Li et al. established a stochastic heroin epidemic model with Lévy jumps [37]. The model was shown to admit a unique global positive solution for any given positive initial value, and the asymptotic behaviors of the solution around the DFE and EE of the corresponding deterministic model were investigated respectively. In 2019, Liu et al. modified the White-Comiskey model into a stochastic heroin epidemic model and investigated the existence and the extinction of ergodic stationary distribution of the model [38]. Similar analyses were applied to another stochastic heroin epidemic model put forward by the same research group in the same year, which differed from the previous one in that a bilinear incidence function was adopted [39]. In the meantime, Wei et al. proposed a stochastically perturbed heroin epidemic model under non-degenerate noises, proved the existence and uniqueness of the global positive solution, and established the unique stationary measure of the system under the non-degenerate case [40]. Moreover, Rafiq et al. presented another stochastic heroin epidemic model and conducted numerical analyses by means of various stochastic explicit or implicit methods [41].

### 3.5. South Africa’s Methamphetamine Models

Apart from the widely discussed heroin epidemic models, the past decade has witnessed a variety of methamphetamine epidemic models. Interestingly, all identified methamphetamine models in this review adopted South African scenarios and were fitted to historical data of patients seeking treatment of methamphetamine abuse. This phenomenon was partially due to the high prevalence of methamphetamine abuse and social burden induced by it in South Africa, especially in the West Cape Province [42,43]. In 2010, Nyabadza et al. proposed the first model of this category, which differed from the aforementioned ones in terms of basic assumptions and model formulation [42]. The model defined two more compartments aside from the ones defined in the classic heroin models, i.e., light drug users and recovered individuals. Besides, a complex incidence function incorporating the impact of behavior changes was adopted, and a sophisticated version was considered for illustrational simulation. As with many other studies, the authors established conditions for threshold dynamics and backward bifurcations of the system and conducted sensitivity analysis as well. However, one of the most innovative attempts is to fit the model to data on treatment demand during 1996–2008 and to make projections on methamphetamine prevalence based on the parameters acquired. The simulation results showed that methamphetamine prevalence in the West Cape Province might have already peaked and was on the decline.

In 2012, Kalula et al. formulated a six-compartment methamphetamine epidemic model based on the previous Nyabadza model [44]. The model considered a core and non-core group, a cycle of light and hard drug users, and a relapse cycle between hard drug users and drug users in treatment as well. The authors obtained conditions for threshold dynamics of the system and investigated the phenomenon of backward bifurcations. Besides, numerical simulation results showed similar trends to that of the previous study, and projection implied that methamphetamine prevalence would decline over a 5-year period so long as the dynamics remained unperturbed. Nyabadza et al. developed a dynamical model of crystal meth abuse in the presence of the drug-supply chain in 2013 [44]. What made this study unique was the incorporation of drug density in the supply chain as a parallel compartment and the application of Latin hypercube sampling (LHS) and partial rank correlation coefficients (PRCC) in sensitivity analyses.

Another three methamphetamine epidemic models were proposed by Mushanyu et al. in 2015, 2016 and 2017, respectively [45,46,47]. The first one considered two types of drug users undergoing treatment, namely, in-patients and out-patients [45]. In-patients were offered shelters and 24-h support, while out-patients resided at home and visited rehabilitation centers on a regular basis. The model was fitted to proportions of methamphetamine users receiving inpatient and outpatient rehabilitation in Cape Town, and the estimated proportion of inpatient rehabilitants would continue to decrease for the next 5 years. Based on the previous model, the second one accounted for saturation effects in the rehabilitation process as well as risk structure in susceptibility [46]. The authors adopted a modified Hill function in rehabilitation rate and restricted themselves to a simplified version for mathematical tractability. Data were obtained from the previous study, and numerical simulations underlined the importance of the capacity of rehabilitation. The last study incorporated the effect of peer influence through a complex incidence function containing a quadratic term and investigated the role of imitation on adolescence methamphetamine use dynamics [47]. After fitting the model to proportions of adolescents aged under 20 reporting methamphetamine as their primary drug of abuse during 2000–2013, simulation results suggested declining proportions and incidence of adolescent methamphetamine users in the near future.

### 3.6. Other Drug Epidemic Models

Quite a few innovative drug epidemic models have emerged ever since the White-Comiskey model, and these models differed from each other in various aspects, including conceptualization, basic assumptions, mathematical forms and application scenarios. Although many of the studies reviewed in this section were parameterized for real settings, few of them were fitted to historical data.

#### 3.6.1. Heroin Epidemic Models

In 2017, Wangari et al. extended the model by Wang et al. to a heroin epidemic model with the recovered group and saturated treatment function [48]. In the presence of backward bifurcation, both the Lyapunov direct method and the geometric approach were adopted in the investigation of global dynamics. The authors conducted sensitivity and uncertainty analysis using LHS in combination with PRCC, and the extent of saturation was identified as one of the key parameters for eradication of the heroin epidemic. An age-structured heroin transmission model with a vaccinated group was formulated by Duan et al. in 2019, and the vaccination effect was assumed to be imperfect and time-variant [49]. Through analysis of the threshold dynamics and basic reproduction number, the authors deduced that a heroin vaccine would definitely be beneficial. In the same year, Memarbashi et al. proposed a heroin epidemic model incorporating the effect of educational prevention programs [50]. The model subdivided the susceptible population into three compartments: the noneducated, educated, and susceptibles who were fully aware of drug harms and would never use it again. The authors qualitatively studied the dynamical behaviors of the system, including the occurrence of backward bifurcation. Furthermore, Abdurahman et al. extended the model by Liu et al. to a heroin epidemic model with two conscious stages and two distributed delays [51]. The model subdivided drug users not in treatment into two categories: the unconscious and conscious ones, representing drug users in the earlier and later stage of addiction, respectively.

#### 3.6.2. Synthetic Drug Epidemic Model

In 2018, Ma et al. proposed a synthetic drug transmission model with psychological addicts and general incidence rate [52]. The authors assumed that a susceptible individual must become psychologically addicted before entering the compartment of physiological addicts and allowed for multiple incidence functions in the initiation process. Within the same year, a methamphetamine epidemic model involving the role of family was formulated by Naowarat et al. [53]. The model adopted a complex incidence function, which was negatively correlated with the effectivity of family influence. Besides, Saha et al. expanded the model by Ma et al. to a synthetic drug transmission model with optimal control [54]. The model adopted Michaelis-Menten (Holling type-II) functional response in the initiation process and further incorporated a saturated treatment function containing a control variable for the analysis of optimal control problem. The authors proved the existence of optimal control function and determined the analytical characterization of the optimal control path.

Liu et al. modified the White-Comiskey model to a synthetic drug transmission model in 2019, by redefining the compartments and adding a group of susceptible individuals with a history of drug abuse [55]. The authors assumed that susceptible individuals who had a history of drug abuse were more likely to become a drug user than those who had not. The study obtained sufficient conditions for the threshold dynamics of the system and conducted sensitivity analysis as well as numerical simulations. This model was revisited by Zhang et al. shortly after, who considered time delays in two rehabilitation processes [56]. The study investigated sufficient conditions for the existence of delay-induced Hopf bifurcation and determined the direction of the Hopf bifurcation as well as the stability of the bifurcating periodic solutions.

#### 3.6.3. Other Modeling Attempts

In 2018, Li et al. formulated a drug transmission model with family education and public health education [57]. The model divided the susceptible population into those with and without health education, and a proportion of new recruits enter these two compartments, respectively. Besides, a transfer process from the susceptible individuals without health education to the educated ones was enabled through public health education. The authors conducted a sensitivity analysis and emphasized the need to combine family education with public health education. In the following year, Su et al. proposed a population-based model and produced estimates of the national trend of drug use in China during 2000–2030 [58]. This model was more akin to a conceptual model, where only three compartments were considered: heroin-only users, synthetic drug-only users and poly-drug users. The model assumed that a drug user must initiate using either heroin or synthetic drug solely before entering the compartment of poly-drug users. Likewise, a poly-drug user had to withdraw from one type followed by the other. The authors conducted an exhaustive survey of existing literature and data sources and fitted the model to the reported numbers of drug users in China during 2000–2017. Projections of the period of 2018–2030 implied the dominance of synthetic drug use, and the permanence of poly-drug use as well.

A coinfection dynamic model of heroin and HIV transmission was designed by Duan et al. in 2020, which might be the first study of this type in the area of drug epidemic models [59]. The five-dimensional model accounted for the compartments of heroin users, HIV-infected individuals, coinfected individuals and those with AIDS. The authors acquired sufficient conditions for the threshold dynamics of two semi-trivial equilibria, which were dependent on two basic reproduction numbers and two invasion reproduction numbers, respectively. Besides, numerical simulations verified the occurrence of a coexistence equilibrium. The model was fitted to data of heroin use and HIV cases in the USA since 2005, and the results suggested a strong coexistence mode.

## 4. Discussion

### 4.1. Overall Features

Apart from the few studies referred to as “early attempts” in Section 3.2, the majority of the studies reviewed in this article were based on nonlinear differential equation systems. The identified drug epidemic models were mostly developed by mathematicians instead of epidemiologists or public health specialists, and a lot of attention was paid to the investigation of the theoretical behaviors of the model systems, including basic reproduction number, feasible region or uniform persistence, local and global asymptotic stability, as well as uncertainty or sensitivity analysis. Some of the studies were simply secondary analyses or extensions of the existing models, such as relaxing assumptions of constant total population, or adding time delays to the initiation or rehabilitation process [19,20,22]. Few of the existing studies were parameterized for real settings or application scenarios, even fewer were fitted to observed historical data [42,43,44,45,46,47].

When it comes to intervention strategies, more than half of the included studies considered both compartmental and parametric interventions (25/48), but most of these models only included a single compartment under rehabilitation, usually named “drug users undergoing treatment”, without specifying which type of treatment strategy or rehabilitation facility was adopted [8,19,20,21,22,23,24]. Besides, parametric interventions were mostly generated through sensitivity analysis of the basic reproduction number, usually involving the normalized forward sensitivity index [60]. Such analyses were able to identify the most effective parameters to curtail the basic reproduction number, and “prevention is indeed greater than cure” was one of the most frequent conclusions reached [8,21,22,26,28]. However, the correlation between specific control measures and the potency in adjusting values of certain parameters has barely been explored.

### 4.2. Utilization of Mathematical Tools

The modeling studies reviewed in this article adopted several advanced mathematical tools to account for complex situations in reality. For instance, time delays or even distributed delays were incorporated in the initiation or relapse processes to imitate the time durations taken to transfer between certain compartments [21,22,23,24]. However, cautions shall be taken when applying such techniques to drug epidemic models, since the introduction of distributed delays will undoubtedly result in a DDE system, for which the analyses of asymptotic behaviors and threshold dynamics are much more complicated than that of regular ODE systems. Moreover, parameterization of these DDE models requires extra data sources, and additional distribution functions are needed for those involving distributed delays [22,23,24]. Search and extraction of the required datasets can be difficult and time-consuming, and total absence of data related to certain populations is not even surprising. Besides, given the complexities of nonlinear dynamic systems and the time span of the simulations, even tiny flaws in a distribution function could result in disastrous consequences. In the case of scarcity of available datasets, field research of the desired parameters might seem impractical for most modelers, and the representativeness of such surveys is open to discussion.

Similar logic also applies to PDE models or nonautonomous systems. Nearly all studies based on PDE models included in this review claimed to incorporate “treat-age”, which was actually the time duration under treatment, in their models [27,28,29]. These models allowed certain parameters to be dependent on treatment duration, such as rehabilitation rate or relapse rate, and the compartments involved showed dual-dependence of time and treatment duration. Prerequisites for the application of this method include well-defined initial conditions and boundary conditions, as well as distribution functions of the age-dependent parameters. Likewise, drug epidemic models based on nonautonomous systems enabled some parameters or coefficients to be time-variant, which frequently took the form of periodic or piecewise functions [21]. Shortage of accurate and up-to-date data sources and imprecision in the formulation of essential distribution functions would both hinder practical applications of these complex models.

In view of the randomness and stochasticity of real life, stochastic drug epidemic models were formulated, which frequently involved white noise or Brownian motion. As shown in the simulation of [39], the long-term results of SDE models tend to fluctuate around the results of the corresponding deterministic model. However, specific forms and intensities of the stochastic terms shall be chosen with caution, since the systems with high stochasticity would show excessive fluctuations in the long run, which is not the case in most practical occasions [1,2,61]. In addition, the theoretical analysis of these systems required advanced theories and techniques, and none of the stochastic models identified was fitted to historical data.

### 4.3. Knowledge Gaps

Modelers of drug epidemic models, who were mostly mathematicians without a medical science background, usually lack the essential knowledge of basic medical concepts and underlying mechanisms. Therefore, chances are that inappropriate assumptions were made when models were formulated without cooperation with epidemiologists or public health experts. For example, Ma et al. considered a compartment of psychological addicts when establishing their synthetic drug transmission model, and the concept was borrowed subsequently by Saha et al. in their model [52,54]. Both studies assumed that a susceptible individual must become psychologically addicted to synthetic drugs before physiological addiction takes place, which might not be supported by existing literature [62,63,64,65,66,67]. Although the theory of addiction mechanism has undergone decades of evolvement, a general consensus has been reached that those psychological symptoms of substance use disorder (SUD) are manifestations of neuropharmacological processes of the central nervous system (CNS) [64,65,66]. In other words, an independent stage of psychological addiction without physiological changes may not exist, and relevant modeling assumptions shall be avoided.

Another bold attempt was made by Duan et al. by incorporating vaccination into their age-structured heroin epidemic model [49]. The authors added a vaccinated group to the model and assumed temporary and imperfect protection against addiction. To the best of our knowledge, however, there is no such thing as drug vaccines available on market for the time being [68,69,70]. Despite decades of research on immunotherapy against SUD and a few promising candidate vaccines in animal models, none has been licensed for clinical application [69,70,71,72]. In view of this, we recommend against using the idea of drug vaccine in drug epidemic modeling studies.

Quite a few studies reviewed in this article allowed for the process of “self-cure” or “self-abstinence” in their models, which means light drug users were able to quit using drugs without any form of treatment [42,43,44,47,48]. We suggest that modelers should think twice about adopting such assumptions since such phenomena as “self-abstinence” can barely be found in published literature [73,74]. Many relevant studies referred to “self-efficacy” in combination with inpatient or outpatient treatment, and successful “self-detoxification” claimed by several articles only lasted a few days, which is negligible compared with the basic time units (1 year or 0.5 year) of regular drug epidemic models [75,76,77,78]. Consequently, processes of “self-abstinence” may seem incompatible for most drug epidemic models.

### 4.4. Perspectives

Based on the discussions above, we strongly recommend that future drug epidemic modeling studies should take the following aspects into consideration: drug type, population definition, data availability and system complexity. According to the World Drug Reports in recent years, the most popular drugs differed greatly among different countries and regions around the globe in terms of type and amount consumed [1,2]. Hence it is of vital importance to choose appropriate drug types and make region-specific assumptions when formulating a drug epidemic model [9]. Given the well-established results of coinfection epidemic models, Duan et al.’s co-transmission model of heroin and HIV was an innovative attempt in this area [59,79,80,81,82,83,84,85,86,87]. Similar issues should be taken care of when designing compartments, since some widely adopted treatment strategies or rehabilitation facilities may not be available or acknowledged in other parts of the world [88]. Besides, even the definition of susceptible populations may vary significantly across settings [42,43,44,45].

Data availability has always been one of the most important issues in epidemic models of any type. Since drug epidemic models involve several hard-to-reach populations, initial values of certain variables and parameters are essentially invisible in real life, which hampered the implementations of parameterization and numerical simulation. The adoption of PDE systems or multi-layered models further complicated this situation, in that distribution functions or inter-layer coefficients were complex in nature and barely accessible [89,90]. For practical purposes, we recommend using piecewise functions as distribution functions, and constant delays instead of distributed delays. These suggestions were raised also for the ease of application, since highly complicated dynamic systems with a huge number of parameters will prevent themselves from massive use.

In this article, we aim to provide an exhaustive investigation and scoping review of all existing literature of drug epidemic models. The search strategy was designed and implemented in accordance with the PRISMA guidelines, but we have to admit the possibility of missing eligible studies from other databases [12]. Moreover, owing to limited space, a thorough explanation of all mathematical concepts and theorems is beyond the scope of this article and hence not included. Despite these limitations, this review comprehensively summarized all drug-epidemic models and give perspectives for further research, which could assist policymakers and public health workers in gaining deeper insights into modeling tools, and help modelers improve their works, thus narrowing gaps between mathematical epidemiology and public health studies.

## 5. Conclusions

We find that the dynamics of the drug epidemic have some specific properties: (1) Its time span is relatively long, and the prevalence of drugs is generally measured in years. (2) In terms of immune mechanisms, there is currently no effective vaccine protection for drug abuse. Moreover, drug addicts currently have a poor prognosis, which means prevention of drug abuse could not depend on biological intervention. (3) From the perspective of high-risk groups, drug epidemics are generally concentrated in a few special groups (MSM, sex workers, drug dealers, etc.), while the general population has less chance of being infected. That is, drug epidemiology is population specificity. (4) There is no incubation period for drug abuse. Once infected, there is a high probability that addiction will gradually increase. (5) Drug epidemic data is scarce and confidential, so it is difficult to construct a detailed model combined with social factors.

We present in this scoping review a thorough investigation of all existing drug epidemic models to date, with the aim of providing an integrated characterization of various advanced mathematical tools to policymakers and public health workers, and helping modelers refine their works in terms of model formulations and parameterization. The majority of the articles reviewed focused on theoretical analyses of the dynamical behaviors of the model systems, and little attention was paid to practical application in real scenarios. We hope that this review will narrow the gaps between mathematical epidemiology and public health research, and recommend that modelers should cooperate with epidemiologists or drug specialists to achieve higher goals in the fight against the drug epidemic.

## Figures and Tables

**Table 1:** Model characteristics and settings of included studies.

Study	Number of Compartment ^a^	Equation Type ^b^	Incidence Function	Intervention	Drug Type	Scenario
Hoppensteadt 1981	2	PDE	bilinear	parameter	-	-
Knolle 1997	2	ODE	linear	-	multiple	Switzerland, 1979–1995
Almeder 2004	2	PDE	complex	parameter	-	-
Caulkins 2007	5	DE	linear	parameter	illicit drug	Australia, 1960–2010
Caulkins 2009	2	ODE	complex	-	-	Australian IDU and US cocaine
Caulkins 2010	2	ODE	complex	-	-	Australian IDU and US cocaine
White 2007	3	ODE	standard	both	heroin	-
Mulone 2009	3	ODE	standard	compartment	heroin	-
Nyabadza 2010	5	ODE	complex	both	methamphetamine	South Africa, 1996–2008
Samanta 2011	3	DDE	bilinear	both	heroin	-
Wang 2011	3	ODE	standard	compartment	heroin	-
Liu 2011	3	DDE	bilinear	both	heroin	-
Kalula 2012	6	ODE	complex	both	methamphetamine	South Africa, 1996–2009
Huang 2013	3	DDE	bilinear	compartment	heroin	-
Nyabadza 2013	6	ODE	complex	both	methamphetamine	South Africa, 1997–2010
Muroya 2014	3	ODE	bilinear	compartment	light drug	-
Abdurahman 2014	3	DE	bilinear	both	heroin	-
Fang 2014	3	DDE	bilinear	both	heroin	-
Fang 2015 ^a^	3	PDE	bilinear	both	heroin	-
Fang 2015 ^b^	3	PDE	bilinear	both	heroin	-
Mushanyu 2015	4	ODE	complex	compartment	methamphetamine	South Africa, 1999–2013
Liu 2016	3n	DDE	complex	compartment	heroin	-
Yang 2016	3n	ODE	complex	both	heroin	-
Yang 2016	3	PDE	complex	both	heroin	-
Mushanyu 2016	5	ODE	complex	both	-	South Africa
Liu 2016	3n	DDE	complex	both	heroin	-
Djilali 2017	3	PDE	complex	both	heroin	-
Wangari 2017	4	ODE	bilinear	both	heroin	-
Mushanyu 2017	4	ODE	complex	both	methamphetamine	South Africa, 2000–2013
Li 2018 ^a^	3	SDE	bilinear	compartment	heroin	-
Ma 2018	4	ODE	complex	both	synthetic drug	-
Li 2018b	6	ODE	bilinear	both	-	-
Naowarat 2018	5	ODE	complex	compartment	methamphetamine	-
Wang 2019	3n	PDE	bilinear	both	heroin	-
Liu 2019	3	SDE	bilinear	both	heroin	-
Liu 2019	3	SDE	standard	both	heroin	-
Duan 2019	4	PDE	bilinear	compartment	heroin	-
Wei 2019	3	SDE	standard	compartment	heroin	-
Su 2019	3	ODE	linear	-	multiple	China, 2000–2030
Memarbashi 2019	5	ODE	standard	compartment	heroin	-
Abdurahman 2019	4	DDE	complex	compartment	heroin	-
Zhang 2019	4	DDE	bilinear	compartment	synthetic drug	-
Liu 2019	3	PDE	bilinear	both	heroin	-
Liu 2019	4	ODE	bilinear	both	synthetic drug	-
Rafiq 2019	3	SDE	bilinear	compartment	heroin	-
Saha 2019	4	ODE	complex	both	synthetic drug	South Africa
Duan 2020	5	ODE	standard	parameter	heroin and HIV	USA, 2005–2017
Duan 2020	3	PDE	bilinear	compartment	heroin	-

^a^ Number of compartments include expressions containing n, which denotes that the model was stratified into n parallel layers. ^b^ Abbreviations: ODE (ordinary differential equations), PDE (partial differential equations), DE (difference equations), DDE (delayed differential equations), SDE (stochastic differential equations). For simplicity, a coupled system of PDE and ODE is referred to as PDE, and the same rule applies to other equation types.

## Data Availability

Not applicable.

## References

[1] UNODC (2019). World Drug Report 2019.

[2] UNODC (2010). World Drug Report 2010.

[3] Greene M.H. (1974). An Epidemiologic Assessment of Heroin Use. Am. J. Public Health.

[4] Jacobs P.E. (1976). Epidemiology Abuse: Epidemiological and Psychosocial Models of Drug Abuse. J. Drug Educ..

[5] Mackintosh D.R., Stewart G.T. (1979). A mathematical model of a heroin epidemic: Implications for control policies. J. Epidemiol. Community Health.

[6] Kermack W.O., McKendrick A.G. (1937). Contributions to the mathematical theory of epidemics IV. Analysis of experimental epidemics of the virus disease mouse ectromelia. J. Hyg..

[7] Rossi C. (2004). Operational models for epidemics of problematic drug use: The Mover–Stayer approach to heterogeneity. Socio-Econ. Plan. Sci..

[8] White E., Comiskey C. (2007). Heroin epidemics, treatment and ODE modelling. Math. Biosci..

[9] Ma Z.E., Zhou Y.C., Wang W.D. (2004). Mathematical Models and Dynamics of Infectious Disease.

[10] Sharareh N., Sabounchi S.S., McFarland M., Hess R. (2019). Evidence of Modeling Impact in Development of Policies for Controlling the Opioid Epidemic and Improving Public Health: A Scoping Review. Subst. Abus. Res. Treat..

[11] Arksey H., O’Malley L. (2005). Scoping studies: Towards a methodological framework. Int. J. Soc. Res. Methodol..

[12] Moher D., Shamseer L., Clarke M., Ghersi D., Liberati A., Petticrew M., Shekelle P., Stewart L.A. (2015). Preferred reporting items for systematic review and meta-analysis protocols (PRISMA-P) 2015 statement. Syst. Rev..

[13] Hoppensteadt F.C., Murray J.D. (1981). Threshold analysis of a drug use epidemic model. Math. Biosci..

[14] Knolle H. (1997). Incidence and prevalence of illegal drug use in Switzerland in the 1980s and early 1990s: An analytical study. Subst. Use Misuse.

[15] Almeder C., Caulkins J.P., Feichtinger G., Tragler G. (2004). An age-structured single-state drug initiation model—Cycles of drug epidemics and optimal prevention programs. Socio-Econ. Plan. Sci..

[16] Caulkins J.P., Dietze P., Ritter A. (2007). Dynamic compartmental model of trends in Australian drug use. Health Care Manag. Sci..

[17] Caulkins J.P., Tragler G., Wallner D. (2009). Optimal timing of use reduction vs. harm reduction in a drug epidemic model. Int. J. Drug Policy.

[18] Caulkins J.P., Feichtinger G., Tragler G., Wallner D. (2010). When in a drug epidemic should the policy objective switch from use reduction to harm reduction?. Eur. J. Oper. Res..

[19] Mulone G., Straughan B. (2009). A note on heroin epidemics. Math. Biosci..

[20] Wang X.Y., Yang J.Y., Li X.Z. (2011). Dynamics of a Heroin Epidemic Model with Very Population. Appl. Math..

[21] Samanta G.P. (2011). Dynamic behaviour for a nonautonomous heroin epidemic model with time delay. J. Appl. Math. Comp..

[22] Liu J.L., Zhang T.L. (2011). Global behaviour of a heroin epidemic model with distributed delays. Appl. Math. Lett..

[23] Huang G., Liu A.P. (2013). A note on global stability for a heroin epidemic model with distributed delay. Appl. Math. Lett..

[24] Fang B., Li X.Z., Martcheva M., Cai L. (2014). Global stability for a heroin model with two distributed delays. Discret. Contin. Dyn. Syst. B.

[25] Muroya Y., Li H.X., Kuniya T. (2014). Complete global analysis of an SIRS epidemic model with graded cure and incomplete recovery rates. J. Math. Anal. Appl..

[26] Abdurahman X., Zhang L., Teng Z.D. (2014). Global dynamics of a discretized heroin epidemic model with time delay. Abstr. Appl. Anal..

[27] Fang B., Li X.Z., Martcheva M., Cai L. (2015). Global asymptotic properties of a heroin epidemic model with treat-age. Appl. Math. Comput..

[28] Fang B., Li X.Z., Martcheva M., Cai L. (2015). Global stability for a heroin model with age-dependent susceptibility. J. Syst. Sci. Complex..

[29] Yang J.Y., Li X.X., Zhang F.Q. (2016). Global dynamics of a heroin epidemic model with age structure and nonlinear incidence. Int. J. Biomath..

[30] Djilali S., Touaoula T.M., Miri S.E.H. (2017). A Heroin Epidemic Model: Very General Non Linear Incidence, Treat-Age, and Global Stability. Acta Appl. Math..

[31] Liu L.L., Liu X.N. (2019). Mathematical Analysis for an Age-Structured Heroin Epidemic Model. Acta Appl. Math..

[32] Duan X.C., Li X.Z., Martcheva M. (2020). Qualitative analysis on a diffusive age-structured heroin transmission model. Nonlinear Anal. Real World Appl..

[33] Liu X.N., Wang J.L. (2016). Epidemic dynamics on a delayed multi-group heroin epidemic model with nonlinear incidence rate. J. Nonlinear Sci. Appl..

[34] Yang J.Y., Wang L.H., Li X.X., Zhang F.Q. (2016). Global dynamical analysis of a heroin epidemic model on complex networks. J. Appl. Anal. Comput..

[35] Liu L.L., Liu X.N., Wang J.L. (2016). Threshold dynamics of a delayed multi-group heroin epidemic model in heterogeneous populations. Discrete Contin. Dyn. Syst. Ser. B.

[36] Wang J.L., Wang J., Kuniya T. (2019). Analysis of an age-structured multi-group heroin epidemic model. Appl. Math. Comput..

[37] Li G.J., Yang Q.G., Wei Y.C. (2018). Dynamics of stochastic heroin epidemic model with lévy jumps. J. Appl. Anal. Comput..

[38] Liu S.T., Zhang L., Xing Y.F. (2019). Dynamics of a stochastic heroin epidemic model. J. Comput. Appl. Math..

[39] Liu S.T., Zhang L., Zhang X.B., Li A. (2019). Dynamics of a stochastic heroin epidemic model with bilinear incidence and varying population size. Int. J. Biomath..

[40] Wei Y.C., Yang Q.G., Li G.J. (2019). Dynamics of the stochastically perturbed Heroin epidemic model under non-degenerate noises. Phys. A Stat. Mech. Appl..

[41] Rafiq M., Raza A., Iqbal M.U., Butt Z., Azam S. (2019). Numerical treatment of stochastic heroin epidemic model. Adv. Differ. Equ..

[42] Nyabadza F., Hove-Musekwa S.D. (2010). From heroin epidemics to methamphetamine epidemics: Modelling substance abuse in a South African province. Math. Biosci..

[43] Nyabadza F., Njagarah J.B., Smith R.J. (2013). Modelling the dynamics of crystal meth (‘tik’) abuse in the presence of drug-supply chains in South Africa. Bull. Math. Biol..

[44] Kalula A.S., Nyabadza F. (2012). A theoretical model for substance abuse in the presence of treatment. S. Afr. J. Sci..

[45] Mushanyu J., Nyabadza F., Stewart A.G. (2015). Modelling the trends of inpatient and outpatient rehabilitation for methamphetamine in the Western Cape province of South Africa. BMC Res. Notes.

[46] Mushanyu J., Nyabadza F., Muchatibaya G. (2016). Modelling Drug Abuse Epidemics in the Presence of Limited Rehabilitation Capacity. Bull. Math. Biol..

[47] Mushanyu J., Nyabadza F., Muchatibaya G., Stewart A.G.R. (2017). On the Role of Imitation on Adolescence Methamphetamine Abuse Dynamics. Acta Biotheor..

[48] Wangari I.M., Stone L. (2017). Analysis of a Heroin Epidemic Model with Saturated Treatment Function. J. Appl. Math..

[49] Duan X.C., Li X.Z., Martcheva M. (2019). Dynamics of an age-structured heroin transmission model with vaccination and treatment. Math. Biosci. Eng..

[50] Memarbashi R., Pourhossieni M. (2019). Global dynamic of a heroin epidemic model. UPB Sci. Bull. Ser. A.

[51] Abdurahman X., Teng Z., Zhang L. (2019). Global dynamics in a heroin epidemic model with different conscious stages and two distributed delays. Int. J. Biomath..

[52] Ma M.J., Liu S.Y., Xiang H., Li J. (2018). Dynamics of synthetic drugs transmission model with psychological addicts and general incidence rate. Phys. A Stat. Mech. Appl..

[53] Naowarat S., Kumat N. (2018). The Role of Family on the Transmission Model of Methamphetamine. J. Phys. Conf. Ser..

[54] Saha S., Samanta G.P. (2019). Synthetic drugs transmission: Stability analysis and optimal control. Lett. Biomath..

[55] Liu P.Y., Zhang L., Xing Y.F. (2019). Modelling and stability of a synthetic drugs transmission model with relapse and treatment. J. Appl. Math. Comp..

[56] Zhang Z.Z., Yang F.F., Xia W.J. (2019). Hopf Bifurcation Analysis of a Synthetic Drug Transmission Model with Time Delays. Complexity.

[57] Li J., Ma M.J. (2018). The analysis of a drug transmission model with family education and public health education. Infect. Dis. Model..

[58] Su S., Fairley C.K., Mao L.M., Nicholas A.M., Jing J., Cheng F., Zhang L. (2019). Estimates of the national trend of drugs use during 2000–2030 in China: A population-based mathematical model. Addict. Behav..

[59] Duan X.C., Li X.Z., Martcheva M. (2020). Coinfection dynamics of heroin transmission and HIV infection in a single population. J. Biol. Dyn..

[60] Arriola L., Hyman J. (2005). Lecture Notes, Forward and Adjoint Sensitivity Analysis: With Applications in Dynamical Systems, Linear Algebra and Optimisation.

[61] Jia Z., Liu Z., Chu P., Jennifer M.M., Cong M., Shi J., Lu L. (2015). Tracking the evolution of drug abuse in China, 2003–2010: A retrospective, self-controlled study. Addiction.

[62] Franken I.H.A. (2003). Drug craving and addiction: Integrating psychological and neuropsychopharmacological approaches. Prog. Neuro-Psychopharmacol. Biol. Psychiatry.

[63] Volkow N.D., Li T.-K. (2004). Drug addiction: The neurobiology of behaviour gone awry. Nat. Rev. Neurosci..

[64] Baler R.D., Volkow N.D. (2006). Drug addiction: The neurobiology of disrupted self-control. Trends Mol. Med..

[65] Hyman S.E., Malenka R.C., Nestler E.J. (2006). Neural Mechanisms of Addiction: The Role of Reward-Related Learning and Memory. Annu. Rev. Neurosci..

[66] Everitt B.J. (2014). Neural and psychological mechanisms underlying compulsive drug seeking habits and drug memories—indications for novel treatments of addiction. Eur. J. Neurosci..

[67] Volkow Nora D., Morales M. (2015). The Brain on Drugs: From Reward to Addiction. Cell.

[68] Shen X.Y., Orson F.M., Kosten T.R. (2012). Vaccines Against Drug Abuse. Clin. Pharmacol. Ther..

[69] Ozgen M.H., Blume S. (2019). The continuing search for an addiction vaccine. Vaccine.

[70] Pravetoni M., Comer S.D. (2019). Development of vaccines to treat opioid use disorders and reduce incidence of overdose. Neuropharmacology.

[71] Anton B., Leff P. (2006). A novel bivalent morphine/heroin vaccine that prevents relapse to heroin addiction in rodents. Vaccine.

[72] Gentry W.B., Rüedi-Bettschen D., Owens S.M. (2009). Development of active and passive human vaccines to treat methamphetamine addiction. Hum. Vaccines.

[73] Zgierska A., Rabago D., Chawla N., Kushner K., Koehler R., Marlatt A. (2009). Mindfulness Meditation for Substance Use Disorders: A Systematic Review. Subst. Abus..

[74] Sofuoglu M., DeVito E.E., Waters A.J., Carroll K.M. (2013). Cognitive enhancement as a treatment for drug addictions. Neuropharmacology.

[75] Noble A., Best D., Man L.-H., Gossop M., Strang J. (2002). Self-detoxification attempts among methadone maintenance patients: What methods and what success?. Addict. Behav..

[76] Day E., Eggen J., Ison J., Copello A., Fazil Q. (2006). Ethnicity and attempts at self-detoxification from opioid drugs. Drugs Educ. Prev. Policy.

[77] Ison J., Day E., Fisher K., Pratt M., Hull M., Copello A. (2006). Self-detoxification from opioid drugs. J. Subst. Use.

[78] Kenney S.R., Bailey G.L., Anderson B.J., Stein M.D. (2017). Heroin refusal self-efficacy and preference for medication-assisted treatment after inpatient detoxification. Addict. Behav..

[79] Blyuss K.B., Kyrychko Y.N. (2005). On a basic model of a two-disease epidemic. Appl. Math. Comput..

[80] Abu-Raddad L.J., Patnaik P., Kublin J.G. (2006). Dual infection with HIV and malaria fuels the spread of both diseases in Sub-Saharan Africa. Science.

[81] Sharomi O., Podder C.N., Gumel A.B., Song B. (2008). Mathematical analysis of the transmission dynamics of HIV/TB coinfection in the presence of treatment. Math. Biosci. Eng..

[82] Roeger L.I.W., Feng Z., Castillo-Chavez C. (2009). Modeling TB and HIV co-infections. Math. Biosci. Eng..

[83] Keeling M.J., Rohani P. (2011). Modeling Infectious Diseases in Humans and Animals.

[84] Lawi G.O., Mugisha J.Y.T., Omolo-Ongati N. (2011). Mathematical model for malaria and meningitis co-infection among children. Appl. Math. Sci..

[85] Mushayabasa S., Tchuenche J.M., Bhunu C.P., Ngarakana-Gwasira E. (2011). Modeling gonorrhea and HIV co-interaction. Biosystems.

[86] Alizon S. (2013). Co-infection and super-infection models in evolutionary epidemiology. Interface Focus.

[87] Mallela A., Lenhart S., Vaidya N.K. (2016). HIV–TB co-infection treatment: Modeling and optimal control theory perspectives. J. Comput. Appl. Math..

[88] Golichenko M., Chu S.K.H. (2018). Human rights in patient care: Drug treatment and punishment in Russia. Public Health Rev..

[89] Feng X.M., Teng Z.D., Zhang F.Q. (2015). Global dynamics of a general class of multi-group epidemic models with latency and relapse. Math. Biosci. Eng..

[90] Li M.-T., Jin Z., Sun G.-Q., Zhang J. (2017). Modeling direct and indirect disease transmission using multi-group model. J. Math. Anal. Appl..

